# Practical Observations on the Diseases of the Urinary Organs, Particularly Those of the Bladder, Prostate Gland, and Urethra. Illustrated by Cases and Engravings

**Published:** 1816-10

**Authors:** 


### PART II.

### COMPREHENSIVE ANALYTICAL REVIEW OF MEDICAL LITERATURE.

"Tros, tyriusve, nobis nullo discrimine agetur."

*Practical Observations on the Diseases of the Urinary Organs, particularly those of the Bladder, Prostate Gland, and Urethra. Illustrated by Cases and Engravings.* By JOHN HOWSHIP, Member of the Royal College of Surgeons in London, and of the Medico-Chirurgical Society. 1 vol. 8vo. pp. 276.

MR. HOWSHIP's name has long been a guarantee for accuracy of observation, fidelity of detail, and acuteness of discrimination. The present work will not detract from this gentleman's well earned reputation: on the contrary, the utility of the facts, and the importance of the subject, will insure this little volume an honourable seat in the library of every medical practitioner, who is anxious to alleviate the sufferings or insure the confidence of his patient.

If the urinary organs give place in rank to some other viscera of the human frame, their diseases are not less frequent, their derangements less distressing, or the consequences less fatal, than the disorders of function or disorganizations of structure in apparently more vital parts. From various causes, our pathological knowledge in these complaints has been retarded in its progress; principally from the concealed situation of most of the parts concerned; the ambiguity of the symptoms; the aversion of the patient to make known his complaints; and the slow march of the morbid phænomena.

Mr. Howship has confined his remarks to those cases which have exclusively fallen under his own observation: if his views, therefore, are more incomplete in the general outline, they are more distinct and faithful copies of nature as far as they go. They are, indeed, such transcripts from nature and truth, as carry the seal of authenticity on their foreheads in the most unequivocal stamp.

The illustrations of these histories are selected from the valuable preparations of Mr. Heaviside. To analyse such a vast body of facts and observations would be impossible in any reasonable compass; and therefore we shall endeavour to present our readers with such a connected *sketch* of the whole, as may prove a most powerful stimulus to place the original in their libraries.

*The Kidney.*—As almost every renal complaint is connected, more or less, with *inflammatory* action, this last should never be lost sight of. Cullen's nosological character of pure inflammation here is well known; but there is no certain diagnostic symptom by which we can always decide on the inflammatory action being idiopathic or symptomatic of a disposition to form calculous matter. The state of the urine, and the past experience of the patient, are our principal guides. When the calculous particles coalesce into masses, they may continue to increase in the kidney or bladder, till their weight or figure cause irritation and a train of distressing consequences. The patient is comparatively fortunate when this takes place in the bladder, for there an operation may be performed; but in the kidney there is little hope.

The most common presentations of calculous matter in the urine are, 1st, in the form of minute crystalline grains, or a brown impalpable powder, consisting of uric acid and a small proportion of the phosphates; 2dly, a white sand, principally composed of the ammoniaco-magnesian phosphate, frequently containing variable proportions of phosphate of lime. Calculi of uric acid will sometimes acquire a considerable size in the kidney, as may be seen in the plate, No. 1. Mr. H. once found an enlarged kidney filled with a compact earthy substance of a yellowish grey colour, and in consistence resembling bird-lime. The ureter was obliterated. While the urine is depositing gravel at the bottom of the vessel, the thick cloud of dense mucous matter demonstrates the state of excitement in the mucous cavities of the kidney; but this increased secretion of mucus is evidently an effort of Nature to lubricate the surfaces of the parts against calculous irritation, and by unloading the vascular system of the gland, to guard against inflammation there. If this irritation is long continued, the secretion will sometimes assume the purulent character, without any abrasion of the secreting surface. But occasionally abscess will take place in the kidney, and if the matter bursts into the pelvis, and escapes with the urine, it is fortunate. Frequently, however, the pus takes a different direction, and although there may be a temporary relief, the case rarely terminates well.

Another consequence of calculous irritation is renal hæmorrhage, which, when moderate, is rather favourable than otherwise. Distention of the kidney sometimes takes place from obstruction in some part of the urinary passages; a remarkable case of which is given by Mr. Howship, bearing some resemblance, though on a smaller scale, to that of Dr. Johnson's, related in the first Number of this Volume.

### Treatment of the foregoing Affections.

1st. *Inflammatory Action in the Kidney.*—Bleeding, warm bath, mild purgatives, emollient glysters, demulcent liquids. Mr. H. proscribes blisters, on account of the hazard of absorption. We have repeatedly employed them in this species of inflammation with great benefit. Where the constitutional powers are weak, local bleedings; fomentations; opiates, especially where there is reason to suspect calculous irritation. At the turn of life, these local affections are often efforts of the constitution to ward off more serious maladies, and ought to be more narrowly watched.

2d. *Calculous Affection of the Kidney.*—Where excess of uric acid prevails, especially where a calculus has formed in the kidney, or is ready to descend from thence into the bladder, the symptoms will often simulate nephritis. The deposition of reddish brown sand, or fine powder of the same colour, in the urine, will demonstrate the difference. The tendency to throw off uric acid is best obviated by magnesia, which corrects those stomach complaints connected with the evolution of gravel or stone. If it be continued beyond the mark, an opposite evil is generated—"a deposition of the phosphates."

"Neither is the production of the white sand materially less distressing to the patient in the irritation it produces, than the evolution of the red gravel." 20.

The alternate vibrations of the beam are now to be watched, and magnesia and muriatic acid are to be alternately had recourse to, as either extreme predominates. Occasionally the mineral acids are objectionable, and then the citric acid is a good succedaneum. Carbonic acid is also useful, not from preventing the formation of the phosphates, but by holding them in solution in the urine. It can be borne in irritable states of the bladder, too, where other acids are inadmissible. In these cases, there is generally a mixture of irritation and increased vascular action of the parts. The irritation is combated by mild regimen — mucilaginous liquids, as decoct. lini, barley water, &c. with saline medicines and opiates. Where the symptoms verge towards inflammation, the proper means of counteracting them are to be employed.

23. — It is needless to say, that in abscess of the kidney there is little chance of recovery. Where it points externally, it may be opened, warning the patient or friends that he is not to be sanguine of relief from the operation.

*Renal Hæmorrhage*, when moderate, is of little consequence; where profuse, or long continued, rest, cool air, some of the acids, vegetable balsams, sedatives. Where there is calculous diathesis, the mineral acids are obviously objectionable, and here the bals. copaibæ is useful. But where the loss of blood is alarming, alum, superacetate of lead and opium, are the surest means of arresting the flow.

*Distention of the Kidney* is sometimes caused by stricture in the urethra, and therefore the state of this passage should be always examined. The most common cause, however, is a calculus arrested in the ureter. In these cases, full doses of opium, or other anodyne, offer the only chance of a temporary relief.

In illustration of the foregoing principles, nine cases are related; a few of which we shall give an outline of, referring to the work itself for a careful perusal of them all.

CASE 1. *Renal Inflammation prelusive of Gout.* — A gentleman, ætat. 40, was attacked with violent pain in the kidney; nausea, vomiting, micturition. Pain not increased by motion; extended along the ureter; numbness in the thigh; tenderness in the testicle; pulse 100, small, rather hard; thirst; urine high coloured, turbid, depositing a small quantity of calculous matter. A silver catheter went readily into the bladder, and drew off an ounce of water. — *Treatment.* Saline draughts, with tinct. opii, quartis horis; leeches to the loins; fomentations. He gradually recovered in a few days, when he was suddenly seized with nausea, vomiting, discharge of thin watery insipid urine, *accompanied with tenderness, pain, redness, and a swelling about the ball of the great toe.* A few glasses of wine were now prescribed daily, with more generous diet, and a light tonic; the foot to be wrapped up in flannel.

"Immediately inflammation came upon the toe, all remaining uneasiness left the loins, and the general health and strength improved." 31.

What would have been the consequence of Dr. Kinglake's plan of treatment in this *first* attack of gout? Some very serious affection of an internal organ most assuredly!

*The second* CASE details the effects of misplaced gout, where the kidney, the stomach, the bladder, the brain suffered. An imperfect manifestation of the gout in the feet and other external parts was encouraged by warmth and stimulating applications, but the constitution was unable to bring on a regular fit, and the determination to important viscera proved ultimately fatal.

CASE 3. *Deposition of Phosphates cured by Muriatic Acid.*—A tailor, ætat. 50, had been twenty years afflicted with gravel. On admission to St. George's Infirmary, he had pain in the kidnies, general uneasiness in the bladder, constant micturition, sharp cutting pain about the neck of the bladder, great straining, hæmorrhage, retraction of right testicle, numbness of thigh. Urine loaded with mucus, depositing *white sand*, with a few particles of red gravel.—*Treatment.* Prohibition of fermented liquors; to take ten drops of muriat. acid thrice a day in water. Under this plan he got quite well in a month, the irritation subsiding some time before diminution of calculous deposition in the urine.

We must pass over the remaining six Cases, many of which are very interesting, but for which we must refer to the work itself.

1. *Bladder.*—*The sympathies of the urinary organs* are well known; but the *modus operandi* of these sympathies is, we think, but very inadequately explained by connection of nerve or similarity of structure. These, however, are the only causes to which we can trace the effects, in the present state of our knowledge.

2. *Irritable Bladder* is accompanied by a sense of uneasiness in that viscus; micturition; often excessive discharge of mucus; occasional tenesmus and straining, with hæmorrhage. These may be occasioned by gravel or sand in the urine, stone in the bladder, irritation or disease of neighbouring parts, disease in the coats of the bladder itself.

3. *Gravel.*—We are unable to ascertain whether the irritation of calculous matter in the bladder is merely mechanical, or whether it changes chemically the properties of the urine; the effect of remedies is therefore very various and uncertain. The state of the urine discharged, the pain in the loins, the derangement of the digestive organs, &c. will indicate calculous matter when present. The *post mortem* appearances "afford a very interesting demonstration of the extent to which the effects of irritation may be occasionally carried in the living body." The most simple effects are, increased vascularity, increased mucous secretion; then comes on in progress, excessive inflammatory action in the inner membrane, with effusion of coagulable lymph, forming a stratum in which the particles of gravel become permanently fixed. Where this expedient of Nature fails in defending the irritable membrane from the sabulous concretions, violent inflammation succeeds, depositing still thicker layers, in default of which an ultimately fatal disease is established in the bladder. In bad constitutions, the diseased coats of the bladder occasionally throw up—

"—a weak, irritable, vascular fungus, projecting into the cavity, disposed to bleed from the slightest cause, and possessing in this respect, as well as others, the characters belonging to cancer."

4. *Irritation from Stone in the Bladder.*—Here exercise is distressing, especially on horseback; the attempts to void urine are often suddenly interrupted by spasmodic action in the bladder, increasing tenfold the severity of the patient's distress. When, at length, the constitution sympathises with the local affection, the consequences are truly formidable: irresistible urgency to pass water; violent straining; expulsion of the intestinal contents; increased vascular action; disordered stomach; and, at last, total failure of the digestive powers, with parched skin, thirst, watchfulness, hectic fever, delirium, death!

There is a considerable inconstancy or uncertainty in the symptoms attendant on calculus in the bladder; since, in many instances of large concretions found after death, no complaint had been made during life. In general, when the stone is lodged near the neck of the bladder, a sense of heat and itching, sometimes almost intolerable, is felt about the external orifice of the urethra. On the other hand, when the stone happens to have a seat at the fundus of the bladder, the pain is referred to the rectum, a striking instance of which is related by Mr. Howship.

5. *Sympathetic Irritation.*—It is well known, that the bladder sympathises with the rectum, the uterus, the urethra. With the rectum sometimes, where there are worms low down there (an instance of which is related); also on the removal of hæmorrhoidal excrescences by ligature, when there is scirrhus or contraction of the gut. With the uterus, in cancer. With the urethra, in gonorrhœa, especially after the use of injections.

Our author justly observes, that the symptoms apparently denoting stone in the bladder are all occasionally equivocal: the least so, perhaps, is the peculiar sensation at the external orifice of the urethra. There being no infallible test then but the sound, the introduction of this instrument must always precede any attempt to remove the calculus by an operation. It sometimes happens, however, that the sound fails in detecting the presence of stone; and Mr. H. thinks, that the elastic gum catheter, without the stilet, conveys a more delicate impression than the metallic instrument. It also occasions less dread and less pain in the introduction, where irritability prevails about the neck of the bladder.

It is well known, that under the operation of certain lithontriptics, particularly alkalies, the symptoms of stone have frequently disappeared, and induced the belief of the calculus having been dissolved. It is equally well known, that this has happened from the stone getting lodged in a sac or pouch. Mr. Howship is disposed to view this occurrence, not as accidental, but as produced by the exhibition of the said alkalies. Alkalies, he observes, check the generation, or at least the separation of *uric acid,* by the kidnies, in consequence of which the urine is rendered less irritating. But this is not all: he conceives, that

"—they possess a remarkable power in diminishing irritability of the bladder, and allaying excitement of that viscus, even where it has proceeded to the extent of inflammation." 77.

The way in which he supposes their exhibition leads to the encystment of the stone is this: that whereas mineral acids, in particular, have the power of promoting the coagulation and contraction of the fibrin of the blood, alkalies, on the contrary, tend to weaken or prevent the act of coagulation, and, in their concentrated state, to dissolve the fibrin subsequent to its having assumed the solid form.

Thus "they principally operate through the medium of the urine, by slowly and gradually abstracting from the inner surface of the urinary bladder a certain proportion of its excitability, diminishing, upon this principle, not only the disposition, but the power also, for contraction and excitement." 80.

While the alkalies thus go on weakening the coats of the bladder, should any part of it have been *previously* weaker than another, it will be more likely to form the sac or pouch for the stone during the expulsion of the urine. If now the alkalies be desisted from, as they generally are after a certain time, the constitution recovers its vigour, the tone of the muscular coat of the bladder improves, and a contraction is excited round the calculus in the cyst, in which it is happily lodged during life. This hypothesis is, to say the least of it, ingenious, and involves a practical question of considerable importance.

We shall pass over four short sections, on paralysis of the bladder, adherent calculus, lithotomy, and the appearances and structure of urinary calculi, giving a short abstract from the latter. The nucleus of a calculus from the bladder is generally formed of uric acid, proving it of *renal* origin; more rarely the nucleus is agglutinated ammoniaco-magnesian phosphate. The most rare is the mulberry calculus, frequently consisting throughout of oxalate of lime. Where an extraneous body forms the nucleus, the surrounding depositions are generally mixtures of the phosphates. Those calculi that are formed of uric acid are distinguished by their red or deep yellow colour; sometimes smooth, but generally rough, warty surface. Those composed of a combination of uric acid, with ammoniaco-magnesian phosphate are of a pale or grey colour—smooth, frequently crystalline surface. Those composed of oxalate of lime are known by the protuberances and inequalities of surface, (whence their name of mulberry calculi) superior compactness, weight, and darker colour.

### Treatment of the foregoing Diseases of the Bladder.

1. *Irritation from Gravel.*—This, as generally dependent on excessive secretion, either of uric acid or the phosphates in the kidney, must be treated in the manner already mentioned, under the head Kidney. When, however, the vesical irritation has attained a certain point, further measures of relief become necessary. These are chiefly, if not entirely, opium, the hip bath, rest, and attention to the bowels. Where opium is objectionable, the ex. hyoscyami may be usefully substituted.

2. *Irritation from Calculus.*—The treatment here is palliative or radical. *Palliative.* Opium, alkalies, such as lime-water, soap, acidulous soda water, caustic alkali, tonics. The *injection* of alkalies into the bladder is useless and pernicious.

The radical, or *operative* treatment is accurately detailed by Mr. Howship; but, for obvious reasons, is passed over in our analysis.

3. *Irritation from Sympathy.*—The treatment here must be regulated by the nature and site of the original disease. When from stricture in the rectum, the bougie, opiates, &c.; when from carcinoma uteri, opium is our principal dependence; when from improperly treated gonorrhœa, we must *undo*, as speedily as possible, what has been *done*. If the discharge be suppressed, the warm bath, and fomentations will tend to its restoration; to which may be occasionally added, bals. copaibæ to the urethra on a bougie; the regular introduction of which last is indispensable, if the preceding inflammation has brought on stricture.

Six cases are related by Mr. H. illustrative of the foregoing remarks. The first is irritable bladder, with cancerous disease, from Mr. Heaviside's Museum. The principal symptoms were micturition, straining, constant irritation at the neck of the bladder, with discharge of viscid mucus, sometimes streaked with blood. The introduction of the bougie detected stricture in urethra, to which caustic was applied, but in vain, the miserable patient died in a few weeks greatly emaciated. The bladder was found completely diseased, in many places disorganised. The coats were, in some places, destroyed; in others, converted into a loose, membranous, fungous, or cancerous structure.

2. *Extreme Irritation of the Bladder from Stricture in the Rectum.*—This was a man, 52 years of age, who was first affected with severe lumbar pains, which at length shot into the left groin; on which, in a month, supervened pain, but not difficulty in making water, the pain about the neck of the bladder progressively increasing till it became almost insupportable. Constipation succeeded.—The introduction of a bougie detected no stricture; but in examining the prostate gland, a firm and most irritable stricture was found in the rectum. This resisted every means of relief, and the poor wretch was worn out with the disease. Several quarts of serum were found in the abdomen; the intestines were thickened, and felt like leather. The omentum felt like cartilage, was irregularly tuberculated, with membranous septa surrounding honey-comb spaces filled with a matter like cream. A scirrhus was found at the pyloric orifice of the stomach. The lower part of the rectum presented a mass of disease. The structure of the bladder was undiseased, but much thickened and contracted in its coats; the cavity scarcely capable of containing a table spoonful of fluid.

3. A mulberry calculus taken from the bladder of a young man, by Mr. Heaviside. It adhered pretty firmly to the neck of the bladder, but was dislodged, and the young man recovered. A beautiful drawing is given of this calculus with its section.

4. *Singularly large calculus voided spontaneously from the urethra of a female.* The patient was an old black woman at Babea, in the Brazils, who brought forth this *hardy* bantling without obstetric aid.—Another victory for Dr. Kinglake!—The calculus is of an oval figure, weighs four ounces six drachms, the section exhibiting a loose crumbly texture, composed of a small proportion of the phosphates with a large one of mucous or animal matter, forming the nucleus of the stone; round which, to a certain extent, the phosphates have been deposited in successive strata. The case bears considerable analogy to that related by Dr. Yelloly, in vol. 6, Med. Chir. Trans.

We shall close the first part of our analysis with the particulars of an interesting case of *fungus hæmatodes* of the bladder.

Ann Burrows, ætat. 57, admitted to relief from St. George's Infirmary, August, 1813, states, that in 1805 she ceased to menstruate, and for five years afterwards enjoyed good health. She then began to feel smarting and pain in making water, which for six months increased, with severe straining, urgent bearings down, and some discharge of blood. For the last three months she has been much distressed with wandering pains in the loins, settling in the hips. An examination, per vaginam, led to nothing satisfactory. A blister was applied to the loins by her own wish, on the removal of which she mentioned a swelling at the lower part of the belly. The tumor was evident and considerable, fixed, firm, and situated in the region of the bladder, immediately above the pubes to which it appeared to adhere most stedfastly. Some idea was entertained that it was a mass of coagulated blood filling up the cavity of the bladder. Although the pains were violent, she never admitted that they had the shooting or burning feel of cancer. The pulse was small and weak, at 120. There was now great pain and distress, constant watching, considerable fever, the tumour stationary. The grumous bloody urine, which had ceased for a few days, began again to flow, with an occasional thin coagulum of blood at the bottom of the vessel. She soon died.

*Sectio cadaveris.* A large, firm, elastic mass was found projecting upwards from the pelvis; to the anterior bony circle of which, it was firmly adherent. The small intestines were partially inflamed, and adherent to the tumour. From each side of the tumour in the pelvis an extensive chain of diseased lymphatic glands passed upwards upon the loins, towards the root of the mesentery. The whole tumour was dissected out of the pelvis; and on examination, the urethra was found to pass into it. A section of the bladder included a portion of the disease, which consisted of soft white pulpy matter, in what appeared originally to have been the cellular membrane connecting the coats of the bladder; the whole mass having the appearance of a congeries of smaller tumours. It varied in thickness from one to three inches. In some places, this white pulpy matter resembled cream, with here and there some adipose substance. The effused blood formed minute masses, deposited, in some places, so near the surface as to shine through the external membrane. Some of these protruded into the cavity of the bladder, and from their vascular surfaces the blood had exuded. The extravasation of blood into the substance of these tumours had taken place to a greater extent in the enlarged lymphatic glands than in the disease of the bladder; but, in other respects, the disease of the bladder, and that in the lumbal glands, was precisely the same.

Mr. H. has not informed us whether this cerebroid substance was miscible with water, or hardened by the acids, as is the case in real fungus hæmatodes.

( To be concluded in our next. )

